# Perceived microaggressions and migrant health in Germany: the role of psychological capital

**DOI:** 10.1186/s40359-025-03920-5

**Published:** 2026-01-07

**Authors:** Adekunle Adedeji, Constance Karing, Saskia Hanft-Robert, Franka Metzner, Sashikala Subedi, Jeongwon Richter, Maria Salomão, RoseAnne Misajon, Fagbemigun Taiwo, Stefanie Witt, Johanna Buchcik, Julia Quitmann, Klaus Boehnke

**Affiliations:** 1 Faculty of Social Work and Childhood Education, Hamburg University of Applied Sciences, Hamburg, Germany; 2 Department of Medical Psychology, University Medical Centre Hamburg-Eppendorf, Hamburg, Germany; 3 Bremen International Graduate School of Social Sciences (BIGSSS), Constructor University, Bremen, Germany; 4 Department of Educational Psychology, Ludwig-Maximilians-Universität, Munich, Germany; 5 Forschungssynthese, Intervention Und Evaluation, Friedrich-Schiller-Universität Jena, Jena, Germany; 6 Faculty of Health, Hamburg University of Applied Sciences, Hamburg, Germany; 7 The Cairnmillar Institute, Melbourne, Australia; 8 Horizon Resource Network E.V., Hamburg, Germany; 9 Division of Health Systems and Public Health, Faculty of Medicine and Health Sciences, Stellenbosch University, Stellenbosch, South Africa

**Keywords:** Microaggression, Quality of life, Personal resources, Social support, Social exchange, Immigrant, Germany

## Abstract

**Background:**

Repeated exposure to microaggressions, subtle and often unintentional forms of discrimination, can undermine the well-being of migrant populations. This study examines the role of psychological capital, comprising hope, self-efficacy, resilience, and optimism, in mediating and moderating the association between perceived microaggressions and health outcomes among migrants in Germany.

**Methods:**

In the cross-sectional study, a total of 858 participants with a migration background residing across different federal states in Germany completed a questionnaire assessing sociodemographic factors, rates of perceived microaggression (BMS-9), psychological capital (PCQ-12), and mental (PHQ-9) and physical health (PHQ-15). Structural equation modelling (SEM) using AMOS 29 was conducted to examine whether psychological capital mediates the association between perceived microaggressions and health outcomes. Mediation was tested using bias-corrected bootstrapped confidence intervals, with model fit evaluated through established indices.

**Results:**

Psychological capital significantly mediated the association between perceived microaggressions and both mental and physical health outcomes. Higher levels of perceived microaggressions were associated with reduced psychological capital, which in turn negatively affected health outcomes. Psychological capital significantly moderated the association between microaggressions and depressive symptoms, with higher psychological capital *intensifying* the link between microaggressions and poorer mental health; however, it did not moderate the association between microaggressions and physical health.

**Conclusion:**

Perceived microaggressions were linked to poorer mental and physical health among migrants, partly through reduced psychological capital. Although psychological capital supported overall well-being, it did not consistently buffer the health impacts of microaggressions. In practice, this means that programs aimed at strengthening resilience, optimism, and self-efficacy should be combined with institutional measures to reduce discriminatory practices in healthcare, education, and the workplace. Policies that recognise microaggressions and promote inclusive, culturally sensitive environments are essential to improving health outcomes and advancing equity for migrants in Germany.

**Supplementary Information:**

The online version contains supplementary material available at 10.1186/s40359-025-03920-5.

## Background

Migrants often navigate complex social landscapes in their countries of residence, where experiences of discrimination and exclusion can shape the overall well-being of some migrant groups. Among these experiences, perceived microaggressions have emerged as a critical area of study in migration research. Microaggressions refer to subtle, often unintentional, forms of discrimination that reinforce social marginalisation [1, 2]. For affected individuals, these microaggressions manifest in various ways, including self-doubt, questioning of their belonging, assumptions about their abilities or cultural background, and exclusion from social or professional opportunities. A substantial body of evidence has demonstrated that repeated exposure to such experiences negatively affects psychological health. Beyond psychosomatic symptoms, microaggressions are consistently associated with increased anxiety and depressive symptoms, reflecting their insidious impact on mental well-being [3–5]. Repeated encounters with microaggressions can heighten stress, reduce self-esteem, and increase risks for mental health disorders. Grouping these outcomes alongside somatic complaints underscores the broad scope of harm associated with microaggressions.

The health situation of migrants in Germany provides a crucial context for examining these dynamics. While many studies report poorer mental health outcomes among migrants compared to native-born populations, findings are not uniform. Some research indicates no significant differences for certain groups [6–8], and others even suggest better outcomes under specific conditions. These divergent findings may be explained by factors such as the degree of social integration and the role of cultural distance, which is closely associated with social rejection and discriminatory treatment in host societies. Such nuances highlight the need for contextualised approaches when examining migrant health.

At the same time, migrants draw on personal and social resources to navigate these challenges. *Psychological capital*, a construct encompassing hope, resilience, optimism, and self-efficacy, represents one such resource [9] defined psychological capital as a core set of psychological capacities that promote adaptive functioning. This can be further situated within broader theories of social and human capital [10], highlighting how individual resources interact with social environments to influence outcomes. Psychological capital has been linked to coping, resilience, and overall well-being, and thus may play a key role in explaining or buffering the health impacts of microaggressions.

The social experiences of migrants in Germany are shaped by structural and interpersonal factors that influence their integration and daily interactions. Germany, as a major destination for migrants within Europe, has witnessed significant demographic shifts due to increased migration flows, particularly from regions such as the Middle East, Africa, and Eastern Europe [11]. While policy frameworks emphasise integration, many migrants report facing barriers in education, employment, and healthcare due to implicit bias and systemic discrimination [12, 13]. Discriminatory experiences, including microaggressions, can contribute to social exclusion and affect the psychological adaptation of some migrant communities [14]. Understanding these experiences is essential for developing policies that foster inclusion and equity.

Migrant health outcomes are significantly shaped by their social experiences, with disparities evident in both physical and mental health indicators. Research indicates that perceived discrimination, including microaggressions, is associated with increased risk for depression, anxiety, and psychosomatic symptoms [5, 15]. In Germany, migrant populations often exhibit disparities in healthcare access, utilisation, and overall health status, with studies highlighting higher rates of chronic stress and lower levels of self-rated health compared to native-born populations [16]. The compounding effects of structural barriers, social exclusion, and discrimination make it imperative to examine coping mechanisms that mitigate these health risks.

Taken together, this study investigates how perceived microaggressions affect both mental and physical health outcomes of migrants in Germany and examines the mediating and moderating role of psychological capital. By integrating mental and somatic dimensions of health and considering the intersecting influences of cultural background and gender, this work seeks to provide a more comprehensive understanding of how subtle forms of discrimination shape migrant well-being.

### The current study

The current study builds on psychological capital, a composite construct encompassing hope, self-efficacy, resilience, and optimism, as a key personal resource. Psychological capital can also be understood within COR Theory as a personal resource that shapes how strongly microaggressions are associated with health outcomes [9, 17, 18]. Conceptually, Psychological capital may serve as both a pathway linking microaggressions to health outcomes and a resource that moderates these associations. Integrating these dual roles within one framework provides a cohesive, resource-oriented explanation of how and when microaggressions relate to health.

Based on the theoretical framework and prior research, the present study tests the following hypotheses:

- H1. Higher levels of perceived microaggressions will be associated with poorer health outcomes, specifically greater depressive symptoms and more severe somatic complaints.
- H2. Psychological capital will mediate the association between perceived microaggressions and health outcomes. Specifically, greater exposure to microaggressions will reduce psychological capital, which in turn will be associated with poorer mental and physical health.
- H3. Psychological capital will moderate the association between perceived microaggressions and health outcomes. In particular, higher levels of psychological capital will buffer the negative impact of microaggressions on depressive symptoms and somatic complaints.

In addition to psychological factors, sociodemographic and socioeconomic characteristics such as age, gender, education, income, marital status, and length of stay in the host country are known to influence health outcomes among migrants [14, 16]. These variables may also shape exposure to microaggressions and access to psychological resources. Controlling for these covariates in the analysis helps to ensure that the observed associations between microaggressions, psychological capital, and health are not confounded by underlying demographic or socioeconomic differences.

### Psychological capital as a mediator: the resource depletion hypothesis

From the perspective of the Conservation of Resources (COR) Theory [18], exposure to chronic stressors, such as perceived microaggressions is associated with the erosion of individuals’ psychological resources. Mediation models propose that microaggressions may be linked to lower levels of self-efficacy, optimism, and resilience, which are core components of psychological capital. Reduced psychological capital is, in turn, associated with poorer mental and physical health. This aligns with stress appraisal theory [19], which posits that when individuals repeatedly encounter stressors (e.g., microaggressions), their available psychological resources may become strained, potentially contributing to poorer well-being.

Empirical support for this pathway comes from studies demonstrating that individuals experiencing discrimination or social exclusion exhibit declines in self-efficacy and resilience, ultimately leading to adverse psychological and physiological outcomes [5, 20]. Thus, we conceptualised psychological capital as an intervening variable through which microaggressions affect health outcomes (X → M → Y).

### Psychological capital as a moderator: the resource buffering hypothesis

Psychological capital can also act as a moderator that shapes the strength of the association between perceived microaggressions and health outcomes. From a COR standpoint, psychological capital reflects a set of personal resources that help individuals preserve well-being when confronted with resource-threatening stressors. When individuals encounter microaggressions, higher psychological capital may help protect or stabilise mental and physical health by supporting adaptive coping and sustaining motivational and self-regulatory processes—capacities reflected in the core components of Psychological capital and in the broader resilience systems described in developmental research [9, 21].

A moderation model assumes that psychological capital does not change as a function of microaggressions but instead determines how strongly microaggressions are associated with health. In this case, psychological capital functions as a protective factor: for individuals with higher levels of psychological capital, the adverse effects of microaggressions on mental and physical health may be attenuated (X × W → Y). Empirical research supports this buffering effect, showing that individuals with high self-efficacy and resilience exhibit lower physiological and psychological stress responses in the face of discrimination [22, 23].

### A dual-model approach: distinguishing between mediation and moderation

Given these theoretical foundations, we propose testing psychological capital in two distinct but complementary models:

1. Mediation Model (X → M → Y): Microaggressions are negatively associated with psychological capital, which in turn worsens mental and physical health.
2. Moderation Model (X × W → Y): Psychological capital serves as a protective factor, buffering the negative impact of microaggressions on health.

By testing these models separately, we can assess whether psychological capital operates as a mediator (a possible causal pathway) or a moderator (a conditioning variable), thereby contributing to a more nuanced understanding of its role in the association between microaggressions and well-being.

## Methods

### Study design

Cross-sectional data were collected from a sample of 858 participants residing in various German federal states. Participants were adults aged 18 to 65 years (M = 30.33, SD = 8.96), including 376 men (43.8%), 445 women (51.9%), 13 non-binary/diverse (1.5%), and 8 participants (0.9%) who did not report their gender. With respect to generational status, 273 participants (31.8%) were second-generation migrants (born in Germany to parents who were migrants), while 585 participants (68.2%) were first-generation migrants. Participants came from diverse cultural backgrounds, including Western Europe, Eastern Europe, the Middle East, North Africa, West Africa, East Africa, Southern Africa, Central Africa, South Asia, and Latin America. The majority reported holding either temporary or permanent residence permits, EU citizenship, or being an asylum applicant. This diverse composition reflects the heterogeneity of migrant communities in Germany, allowing for broad insights into the association between perceived microaggressions, psychological capital, and health outcomes. Informed consent was obtained from all participants before their participation. The Ethics Committee of the Bremen International School of Social Science granted ethical approval for the study, and all procedures adhered to the guidelines for ethical research.

Data collection took place between March 2023 and December 2023 using an online survey administered via LimeSurvey. This ensured accessibility in seven different Languages (German, English, Arabic, Polish, Russian, Turkish, and Persian) to accommodate the linguistic diversity of the target population. Participants were recruited using purposive sampling, ensuring a structured and systematic recruitment process. To incentivise participation, individuals who completed the questionnaire received a € 5 Amazon gift card as a token of appreciation.

### Measures

#### Predictor variable—perceived microaggressions

Perceived microaggressions were measured using the Beliv Microaggression Scale-9 (BMS-9), a shortened version of the BMS-27, designed to assess the frequency of microaggressions experienced in daily life [24]. The BMS-9 captures perceived microaggressions across three core categories: microinsults, microassaults, and invalidation, each covering three manifestations: verbal, behavioural, and structural. Participants rated nine items on a 5-point Likert scale (0 = never to 4 = very often), indicating the frequency with which they experienced different forms of microaggressions. The microinsult subscale (3 items) captures subtle, usually unintentional comments that stigmatise or demean individuals. An example item is: “How often do you experience the following because of your migration background: Statements, comments, or questions from people that unintentionally stigmatise or hurt you?” The microassault subscale (3 items) assesses overt discriminatory statements or behaviours that communicate inferiority, such as: “How often do you experience the following because of your migration background: Statements, comments, or questions from people that communicate that you are of lesser worth?” The invalidation subscale (3 items) evaluates experiences where one’s identity or lived experiences are trivialised or denied, e.g., “How often do you experience the following because of your migration background: Verbal statements that belittle, trivialise, or deny your experiences?”. A total perceived microaggression score was calculated by summing the responses across the nine items, yielding a total score ranging from 0 to 36, with higher scores indicating a greater frequency of perceived microaggressions. The BMS-9 demonstrated good internal consistency in the current sample, with a Cronbach’s alpha of 0.89, indicating strong reliability.

#### Mediating variable – psychological capital

Psychological capital was assessed using the Psychological Capital Questionnaire (PCQ-12), a 12-item self-report measure developed to evaluate four key psychological resources: hope, self-efficacy (also referred to as efficacy), resilience, and optimism [9]. Each component is measured using three items, and responses are recorded on a 4-point Likert scale (1 = strongly disagree to 4 = strongly agree), with higher scores indicating greater levels of psychological capital.

The hope subscale (3 items) assesses individuals' ability to persevere toward goals and generate pathways to achieve them. An example item is: “I can think of many ways to reach my current goals.” The efficacy (self-efficacy) subscale (3 items) measures confidence in one’s ability to mobilise motivation and resources to accomplish tasks, such as: “I feel confident analysing a long-term problem to find a solution.” The resilience subscale (3 items) evaluates the ability to recover from adversity, setbacks, or uncertainty, with an example item: “I can get through difficult times at work because I’ve experienced difficulty before.” The optimism subscale (3 items) captures positive expectations for the future, as reflected in: “I always look on the bright side of things regarding my job.”. A total psychological capital score was calculated by summing the responses across the 12 items, yielding a total score ranging from 12 to 48. The PCQ-12 demonstrated strong internal consistency in the current sample, with a Cronbach’s alpha of 0.88, indicating high reliability.

#### Outcome variables

##### Mental health

Mental health was operationalised as depressive symptoms, assessed using the Patient Health Questionnaire-9 (PHQ-9), a widely validated instrument frequently employed as an indicator of overall mental health in population-based research [25]. The PHQ-9 evaluates the frequency of depressive symptoms over the past two weeks based on the DSM-IV criteria for major depressive disorder. Participants rated each item on a 4-point Likert scale (0 = "not at all" to 3 = "nearly every day"), resulting in a total score ranging from 0 to 27, with higher scores indicating greater depressive symptom severity.

The PHQ-9 captures symptoms related to anhedonia, depressed mood, sleep disturbances, fatigue, appetite changes, feelings of worthlessness or guilt, concentration difficulties, psychomotor changes, and suicidal ideation. An example item is: “Over the last two weeks, how often have you had little interest or pleasure in doing things?”. Total scores were categorised based on standard severity cut-offs, where scores between 0 and 4 indicate minimal or no depression, 5 to 9 suggest mild depression, 10 to 14 correspond to moderate depression, and 15 to 19 reflect moderately severe depression. Scores of 20 to 27 indicate severe depression. A total PHQ-9 score was calculated by summing the responses across all nine items, with higher scores indicating a greater severity of depressive symptoms. The PHQ-9 demonstrated strong internal consistency in the current sample, with a Cronbach’s alpha of 0.90, indicating excellent reliability.

##### Physical health

Physical health was operationalised as somatic symptom burden using a subset of six items from the Patient Health Questionnaire-15 (PHQ-15) [26]. Specifically, participants rated the frequency of common physical symptoms (e.g., headaches, back pain, stomach or bowel problems, dizziness) over the past four weeks on a standardised Likert scale ranging from "not at all" (0) to "a lot" (2). This reduced item set was used to capture general physical symptom burden while minimising participant burden in a large, multilingual survey. The six-item subset does not represent a formally validated short form of the PHQ-15; however, it demonstrated acceptable internal consistency in the current sample (Cronbach’s α = 0.75). Higher scores indicate greater physical symptom burden.

#### Covariates

The current analysis included sociodemographic and socioeconomic variables as covariates to account for their potential influence on mental and physical health outcomes. Age and duration of stay in Germany were measured as continuous variables, while gender was categorised as male, female, diverse, or other. Educational attainment was classified according to the International Standard Classification of Education (ISCED), ranging from 1 (less than lower secondary education) to 6 (higher tertiary education, including a master’s degree or higher). The region of origin was classified into Western Europe, Eastern Europe, the Middle East, North Africa, West Africa, East Africa, Southern Africa, Central Africa, South Asia, and Latin America. Migration status was categorised as first-generation migrant (born abroad) and second-generation migrant (born in Germany with at least one parent born abroad). Income level was aligned with national income distribution statistics, and family status was classified as single, married/partnered, separated/divorced, or widowed.

### Statistical analysis

Statistical analyses were conducted using SPSS 29 with the PROCESS macro (Version 5.0) by Hayes (2018) for moderation analyses and AMOS 29 for structural equation modelling (SEM) to examine mediation effects. Descriptive statistics and Pearson correlation analyses were first performed to explore the associations between perceived microaggressions, psychological capital, mental health, physical health and sociodemographic and socioeconomic variables. This step provided an initial assessment of variable associations and identified potential concerns regarding multicollinearity.

To assess whether psychological capital mediates the association between perceived microaggressions (X) and health outcomes (Y1 = mental health; Y2 = physical health), a structural equation modelling (SEM) approach was implemented using AMOS 29. A latent variable approach was used to model psychological capital, incorporating observed indicators of hope, self-efficacy, resilience, and optimism [9]. Model fit was evaluated using multiple indices based on established cutoffs [27, 28] chi-square (χ^2^/df), with values below 3 indicating a good fit; the Comparative Fit Index (CFI) and Tucker-Lewis Index (TLI), both of which should be above 0.90; the Root Mean Square Error of Approximation (RMSEA), where values of 0.06 or lower indicate a good fit; and the Standardized Root Mean Square Residual (SRMR), where values below 0.08 are acceptable. The indirect effect of perceived microaggressions on health outcomes via psychological capital was tested using bias-corrected bootstrapped confidence intervals (CI) with 1,000 resamples [29]. Mediation was considered statistically significant if the 95% CI did not include zero [30].

**Fig. 1:**
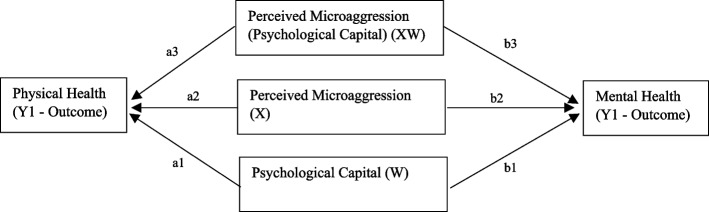
Model examining psychological capital as a moderator of the association between perceived microaggression and physical health, and perceived microaggression and mental health

To examine whether psychological capital moderates the association between perceived microaggressions and health outcomes, a moderation analysis (Model 1 in PROCESS) was conducted [31]. An interaction term (X × W) was computed after mean-centring the predictor (perceived microaggressions) and moderator (psychological capital) to reduce multicollinearity (see Fig. 1 above). A significant interaction effect would indicate that the association between microaggressions and health outcomes varies across different levels of psychological capital. The Johnson-Neyman (JN) technique was applied to further probe significant interactions to determine the regions of significance for psychological capital as a moderator [32]. Simple slope analyses were also performed at low (−1 SD), mean, and high (+ 1 SD) levels of the moderator to visualise interaction effects [33].

Several statistical assumptions and checks were conducted to ensure the robustness of the findings. Heteroskedasticity-consistent standard errors (HC3) were used in PROCESS to account for potential violations of homoscedasticity [34]. Variance inflation factors (VIFs) and tolerance values were examined to assess multicollinearity, and normality was checked using skewness and kurtosis values (acceptable range: ± 2; [28]. Missing data were handled using Full Information Maximum Likelihood (FIML) in AMOS, which has been shown to minimise bias in parameter estimates [35]. Statistical significance was set at *p < *0.05, and effect sizes were interpreted based on guidelines from [36], where values below 0.1 indicate a small effect, values between 0.1 and 0.3 indicate a moderate effect, and values above 0.3 indicate a large effect.

## Results

### Sample characteristics

Participants were adults aged 18 to 65 years (M = 30.33, SD = 8.96). As shown in Table 1, the sample consisted of 855 participants, with a slight majority of females (51.9%) compared to males (43.8%). A small proportion identified as diverse (1.5%) or did not report their gender (0.9%). Nearly half of the respondents (49.3%) were single, and 24.1% were married. A majority of participants were first-generation migrants (69%), while 31% were second-generation. The most represented regions of origin were Sub-Saharan Africa (22.1%), Eastern Europe (16.0%), and the Middle East/North Africa (11.8%). Regarding income, 27.5% reported earning between €521 and €1,500, and 34.6% of responses were missing.

**Table 1:** Sample Sociodemographic and economic characteristics

Variables	Frequency (N)	Percentage (%)
Gender		
Male	376	43.8
Female	445	51.9
Diverse	13	1.5
No answers	8	0.9
Marital Status		
Single	423	49.3
In partnership, but not married	166	19.3
Married, but living separately	21	2.4
Married	207	24.1
Divorced	20	2.3
Widowed	3	0.3
Length of Stay in Germany (Year)		
< 1	45	5.2
1–3	92	10.7
4–5	69	8.0
6–10	142	16.6
> 10	237	27.6
Missing (Born in Germany)	273	31.8
Migrants Group		
First generation	592	69
Second Generation	266	31
Income		
I do not have any income	36	4.2
Less than or equal to €520	64	7.5
€521 to €1,500	236	27.5
€1,501 to €2,500	75	8.7
€2,501 to €3,500	50	5.8
€3,500 and above	54	6.3
Don't know	7	0.8
I prefer not to answer	39	4.5
Missing	297	34.6
Migrant origin		
Eastern Europe	137	16.0
Northern Europe	14	1.6
Central Europe	35	4.1
Southern Europe	48	5.6
Western Europe	22	2.6
North America	14	1.6
South/Central America	36	4.2
Middle East/North Africa	101	11.8
Sub-Saharan Africa	190	22.1
Russia/Central Asia	68	7.9
East Asia	37	4.3
South Asia	95	11.1
Southeast Asia/Australia	47	5.5
Missing	14	1.6

**Fig. 2:**
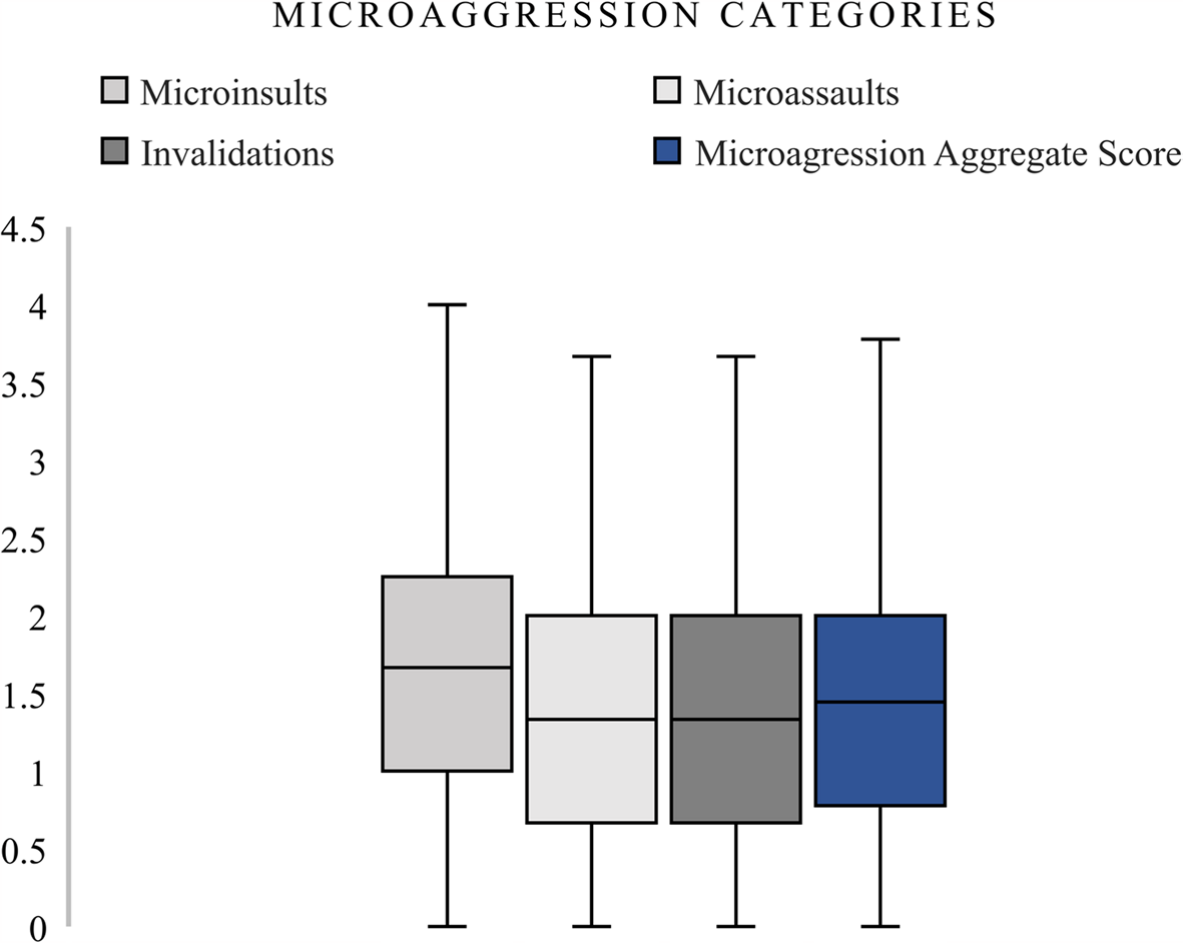
Microaggression Categories

**Fig. 3:**
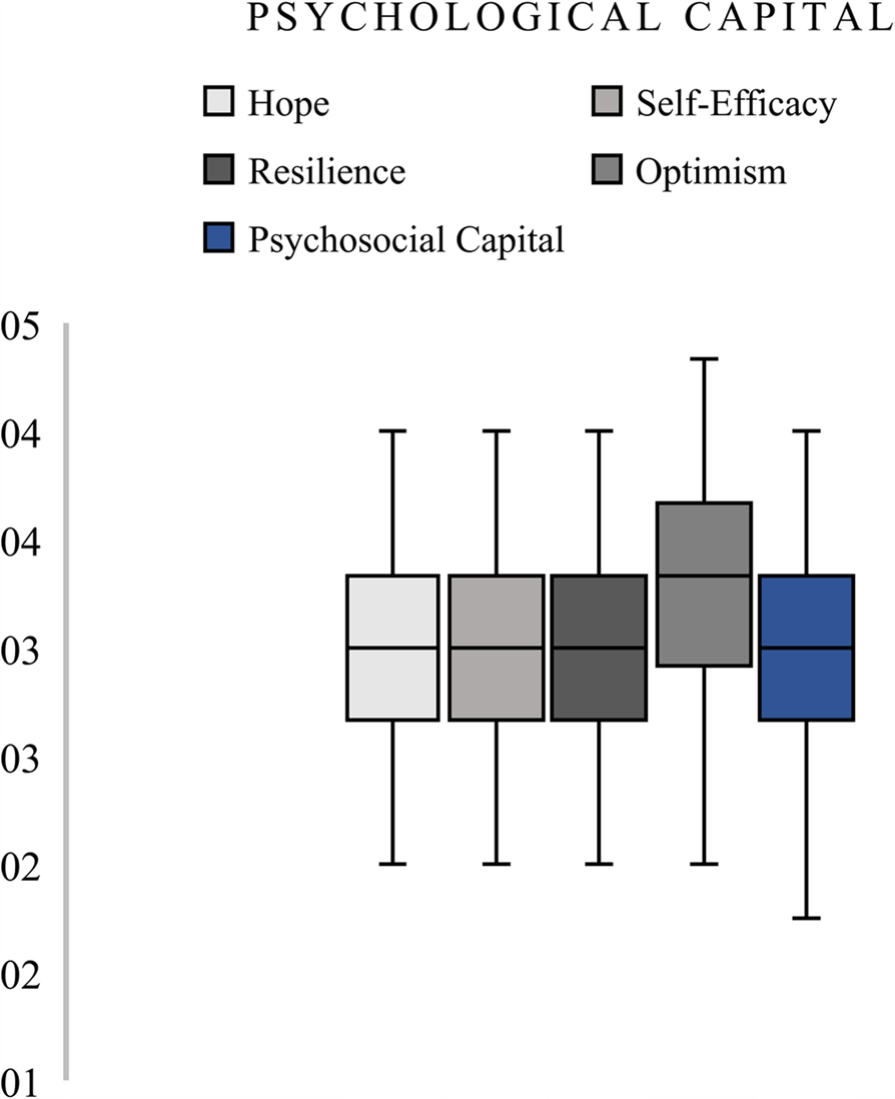
Psychological Capital

Physical health problem scores increased with higher levels of depression severity, with participants reporting severe and moderately severe depression also exhibiting higher physical health problem scores. Figure 2 compares distributions across microaggression subtypes. Microinsults appeared to have slightly higher median frequencies than microassaults and invalidations. The aggregate microaggression score had a broad distribution, reinforcing the prevalence of multiple forms of subtle discrimination among participants. Figure 3 displayed the distribution of psychological capital dimensions. Optimism had the highest median and range, suggesting that participants generally scored higher in this dimension compared to hope, resilience, or self-efficacy. The composite psychological capital score fell near the centre of the distribution, reflecting a balanced representation of the individual psychological resources.

### Group differences by migrational background

A series of one-way ANOVAs examined differences across migrant groups in mental health, perceived microaggressions, physical health, and psychological outcomes (see Table 6 in the Appendix). Perceived microaggressions significantly differed by migrant origin (*p < *0.001), with participants from the Middle East/North Africa (M = 15.62, SD = 7.68) and Sub-Saharan Africa (M = 15.13, SD = 6.32) reporting the highest levels. Mental health also varied significantly among groups (*p =* 0.02), with participants from the Middle East/North Africa reporting the highest mean scores (M = 11.10, SD = 7.00).

Significant group differences were also found for self-efficacy (*p =* 0.03) and optimism (*p =* 0.04), with the lowest self-efficacy reported by participants from North America (M = 7.79, SD = 1.81), and the highest optimism reported by those from East Asia (M = 10.08, SD = 1.92). Hope levels were significantly lower among participants from South Asia (M = 8.13, SD = 1.97; *p < *0.001). No statistically significant group differences were observed in psychological capital or physical health.

### Correlation matrix

A Pearson correlation analysis examined the associations between demographic variables (income, education, age, length of stay in Germany, poorer mental health (as indicated by depressive symptoms), poorer physical health (as indicated by somatic complaints), psychological factors (hope, self-efficacy, resilience, optimism, and psychological capital), and perceived microaggressions. Among the demographic variables, education (in years of schooling) was negatively correlated with poorer physical health (*r =* −0.094, *p < *0.05), suggesting that individuals with higher education reported slightly better physical health. Length of stay in Germany was negatively correlated with poorer mental health (*r =* −0.082, *p < *0.05), indicating that a longer stay in Germany was associated with better mental health. Income was positively correlated with both education (*r =* 0.097, *p < *0.05) and age (*r =* 0.318, *p < *0.01), suggesting that older individuals and those with higher education tend to have higher incomes (see Table 2).

**Table 2:** Pearson correlation matrix of participants’ sociodemographic factors, microaggression and poor health outcomes

		1	2	3	4	5	6	7	8	9	10	11	12	M	SD
1	Poor Mental Health	–												9.76	6.33
2	Poor Physical Health	.578^**^	–											3.93	3.07
3	Hope	-.244	-.347^**^	–										8.67	1.84
4	Self-efficacy	-.204^**^	-.312^**^	.617^**^	–									8.69	1.81
5	Resilience	-.202^**^	-.319^**^	.491^**^	.616^**^	–								8.76	1.89
6	Optimism	-.208^**^	-.335^**^	.491^**^	.502^**^	.515^**^	–							9.54	1.97
7	Psychological Capital	-.265^**^	-.415^**^	.799^**^	.841^**^	.813^**^	.784^**^	–						35.65	6.08
8	Microaggression	.234^**^	.289^**^	-.111^**^	-.105^**^	-.054	-.119^**^	-.20^**^	–					12.74	7.68
9	Income	-.189^**^	-.084^*^	.147^**^	.095^*^	.031	.058	.105^*^	-.074	–				3.76	1.76
10	Education (Years)	-.070	-.094^*^	.036	.033	.033	.013	.040	.018	.097^*^	–			13.23	3.47
11	How old are you?	-.043	-.025	.044	.006	.023	.025	.030	.025	.318^**^	.138^**^	–		30.33	8.96
12	Length of stay	-.082^*^	.041	.057	.013	.026	-.008	.024	-.026	.186^**^	.058	.485^**^	–	11.99	10.82

^**^. Correlation is significant at the 0.01 level (2-tailed). *. Correlation is significant at the 0.05 level (2-tailed)

### Measurement model

**Fig. 4:**
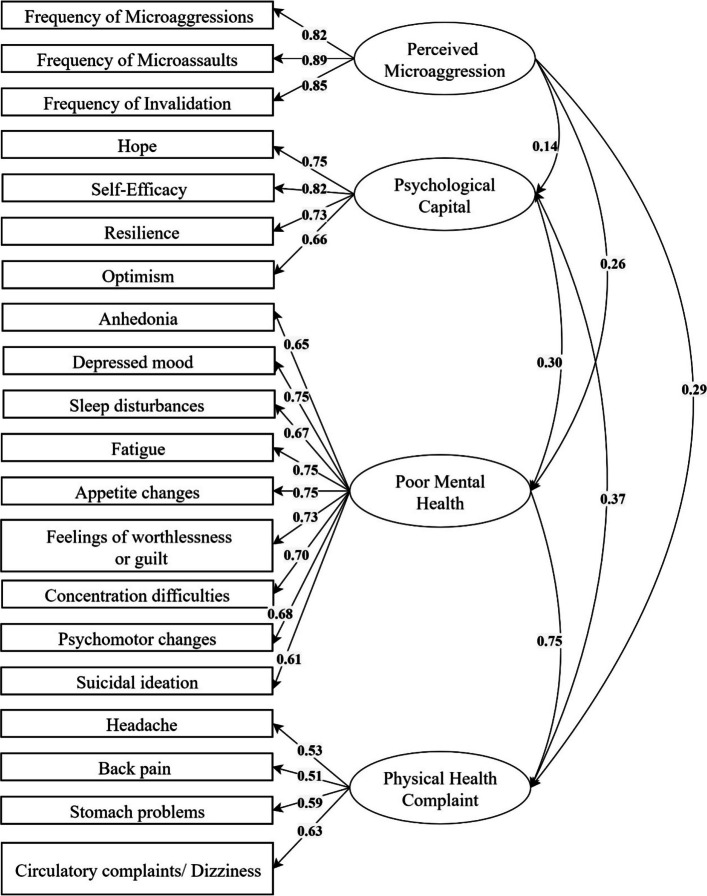
Measurement model perceived microaggression, psychological capital, poor mental and physical health

The conceptual model was assessed using structural equation modelling (SEM) (Fig. 4), and the fit indices indicated an acceptable model fit. The chi-square statistic was significant, χ^2^(164) = 548.07, *p < *0.001, with a chi-square ratio (CMIN/DF) of 3.34, indicating a moderate fit [27]. The comparative fit indices demonstrated good model fit, with NFI = 0.923, RFI = 0.901, IFI = 0.945, TLI = 0.929, and CFI = 0.944, all of which exceed the conventional threshold of 0.90, indicating strong model performance [36]. The RMSEA was 0.052 (90% CI: [0.047, 0.057]), with a probability of close fit (p-close) = 0.214, further supporting the model’s adequacy [37]. The AIC value for the default model (680.07) was lower than that of the independence model (7157.33), suggesting that the proposed model provides a better fit.

The conceptual model, as illustrated in Fig. 4, reveals significant relationships among the constructs. Psychological capital was a strong predictor of self-efficacy (*β* = 0.819), hope (*β* = 0.730), resilience (*β* = 0.734), and optimism (*β* = 0.655), suggesting that psychological resources play a crucial role in fostering positive psychological outcomes. Mental health was well represented by its observed indicators, with standardised factor loadings ranging from 0.606 to 0.755, indicating strong measurement reliability. Similarly, physical health was well captured by its indicators, with factor loadings ranging from 0.510 to 0.634. Microaggressions also formed a cohesive construct, with factor loadings of 0.821 for microinsult, 0.886 for microassault, and 0.845 for invalidation.

Correlations between constructs revealed notable associations. Mental and physical health were positively correlated (*r =* 0.749), suggesting a strong association between mental and physical well-being. Psychological capital was negatively correlated with both psychological distress (*r =* −0.304) and poor physical health (*r =* −0.374), indicating that higher psychological resources are associated with lower distress and better perceived physical health. Microaggressions were negatively correlated with psychological capital (*r =* −0.139), while showing positive correlations with both physical health (*r =* 0.292) and psychological distress (*r =* 0.256). This suggests that greater exposure to microaggressions is linked to higher distress and poorer physical health outcomes.

### Structural mediation model

A structural equation modelling (SEM) analysis was conducted using AMOS 29 to examine psychological capital as a mediator of the associations between perceived microaggressions, mental health, and physical health. The model demonstrated a good fit based on conventional fit indices (CFI = 0.944, TLI = 0.929, RMSEA = 0.052, χ^2^/df = 3.34), indicating an acceptable representation of the data structure.

**Fig. 5:**
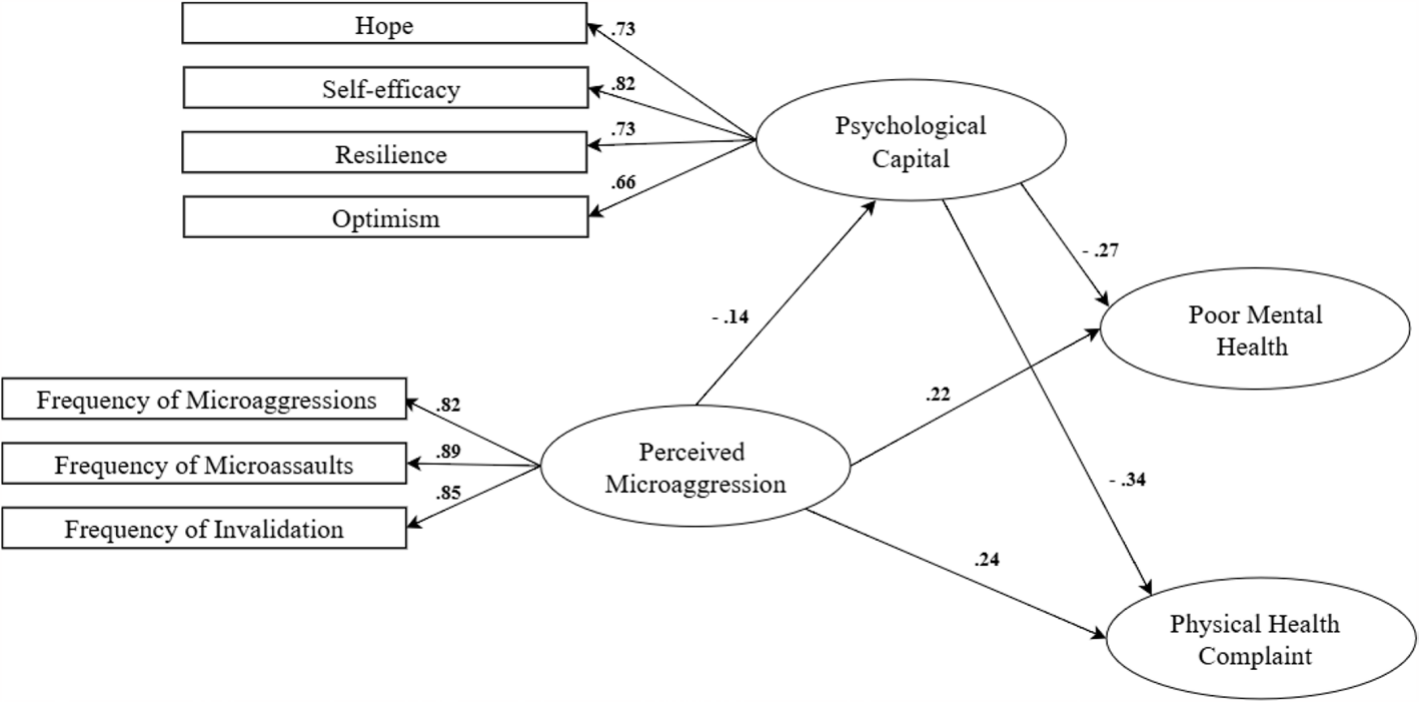
Structural Model: Mediating effect of psychological capital on the association between perceived microaggression and poor health outcomes

As shown in Fig. 5, the regression analysis revealed that perceived microaggressions significantly predicted psychological capital (B = −0.092, SE = 0.026, *p < *0.001, β = −0.139), indicating that higher levels of perceived microaggressions were associated with lower psychological capital. In turn, psychological capital significantly predicted mental health (B = −0.129, SE = 0.019, *p < *0.001, β = −0.274) and physical health (B = −0.090, SE = 0.015, *p < *0.001, β = −0.340), suggesting that individuals with greater psychological capital reported better mental and physical health outcomes. Additionally, perceived microaggressions directly predicted mental health (B = 0.068, SE = 0.012, *p < *0.001, β = 0.218) and physical health (B = 0.043, SE = 0.009, *p < *0.001, β = 0.244), indicating that higher perceived microaggressions were associated with poorer health outcomes.

Among the psychological capital components, psychological capital significantly predicted self-efficacy (B = 1.000, β = 0.819), hope (B = 0.906, SE = 0.044, *p < *0.001, β = 0.730), optimism (B = 0.868, SE = 0.047, *p < *0.001, β = 0.655), and resilience (B = 0.936, SE = 0.045, *p < *0.001, β = 0.734). These results support the conceptualisation of psychological capital as a composite construct contributing to health and well-being. An indirect effects analysis demonstrated that perceived microaggressions influenced mental health (β = 0.038, *p < *0.001) and physical health (β = 0.047, *p < *0.001) through psychological capital, supporting psychological capital as a partial mediator (see Table 3).

**Table 3:** Mediating effects of psychological capital on poor mental and physical health

Predictor (X)	Mediator (M)	Outcome (Y)	Indirect Effect (Unstandardized)	Standardised Indirect Effect	*p*
Microaggressions	Psychological Capital	Physical Health	**0.008**	**0.047**	0.001
Microaggressions	Psychological Capital	Mental Health	**0.012**	**0.038**	0.001

### Psychological capital as a moderator of the association between perceived microaggression and health outcome

A moderation analysis was conducted to examine whether Psychological Capital moderates the association between Perceived Microaggression and Depressive Symptoms (PHQ-9). As shown in Table 4, the overall model was statistically significant, F(3, 837) = 41.63, *p < *0.001, with an adjusted R^2^ of 0.112, indicating that the predictors explain approximately 11.2% of the variance in PHQ-9 scores. The regression results show that Perceived Microaggression did not significantly predict PHQ-9 scores, B = −0.14, t (837) = −0.90, *p =* 0.367. However, Psychological Capital was a significant negative predictor of PHQ-9 scores, B = −0.36, t(837) = −6.02, *p < *0.001, indicating that higher levels of Psychological Capital are associated with lower depressive symptoms. Notably, the interaction between Perceived Microaggression and Psychological Capital was significant, B = 0.0085, t(837) = 2.03, *p =* 0.043, suggesting that Psychological Capital significantly moderates the association between Perceived Microaggression and Depressive Symptoms, but in an amplifying rather than a buffering direction.

**Table 4:** Moderating effects of psychological capital on the association between perceived microaggression and poor mental health

	B	Std. Error	T	Sig
***Constant***	20.446	2.248	9.095	.00
Perceived Microaggression	-.139	.154	-.903	.37
Psychological Capital	-.356	.059	−6.022	.00
***Interaction*** Perceived Microaggression *Psychological Capital	.009	.004	2.027	.04

A Johnson-Neyman analysis revealed that the effect of Perceived Microaggression on PHQ-9 was significant for individuals with Psychological Capital scores above 27.13, meaning that among individuals with higher Psychological Capital, Perceived Microaggression was positively associated with Depressive Symptoms. These findings suggest that while Psychological Capital is generally protective against depressive symptoms.

**Fig. 6:**
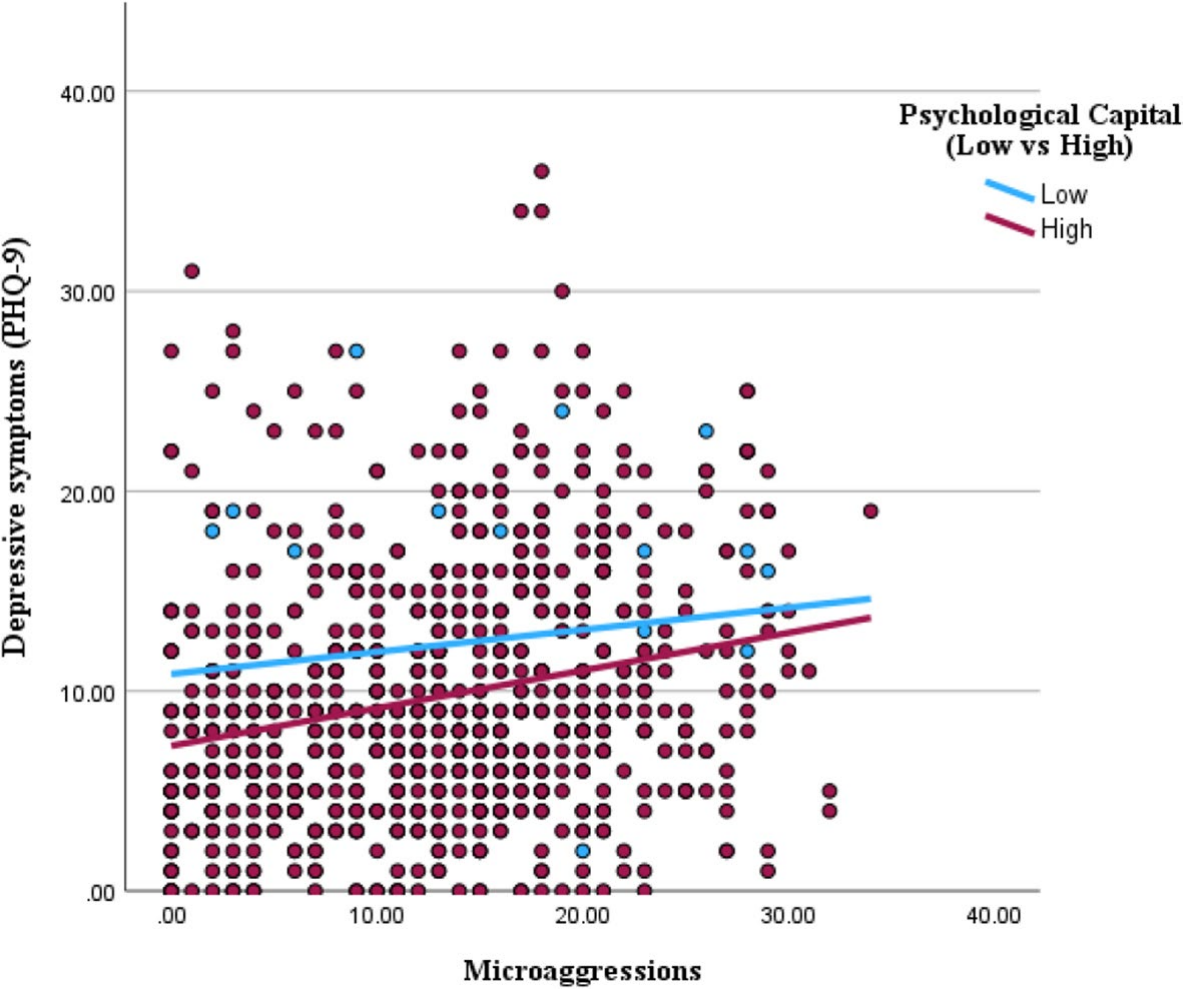
Interaction of Microaggressions and Psychological Capital in Predicting Depressive Symptoms

Figure 6 illustrates the significant interaction between perceived microaggressions and psychological capital in predicting depressive symptoms. Simple slopes analysis showed that microaggressions were not significantly related to depressive symptoms among individuals with low psychological capital. In contrast, among those with higher psychological capital, microaggressions were positively associated with depressive symptoms. This pattern suggests that psychological capital, while generally protective, amplified the adverse impact of microaggressions on mental health at moderate to high levels.

A moderation analysis was conducted using the PROCESS macro (Model 1; Hayes, 2018) to examine whether psychological capital moderates the association between perceived microaggressions and physical health (see Table 5). The overall model was statistically significant, F (3, 563) = 53.20, *p < *0.001, accounting for 22.09% of the variance in physical health (R^2^ = 0.2209). Results indicated that perceived microaggressions significantly predicted physical health (B = 0.16, SE = 0.08, t = 2.03, *p =* 0.043), suggesting that higher levels of perceived microaggressions were associated with worse physical health outcomes. Psychological capital was also a significant predictor (B = −0.17, SE = 0.03, t = −5.02, *p < *0.001), indicating that greater psychological capital was associated with better physical health.

**Table 5:** Moderating effects of psychological capital on the association between perceived microaggression and poor physical health

	B	Std. Error	t	Sig
***Constant***	8.8297	1.2229	7.2202	.000
Perceived Microaggression	0.1626	0.0802	2.0279	.043
Psychological Capital	−0.1659	0.0330	−5.0205	.000
***Interaction*** Perceived Microaggression *Psychological Capital	−0.0021	0.0022	−0.9569	.339

The interaction term between perceived microaggressions and psychological capital was not statistically significant (B = −0.0021, SE = 0.0022, t = −0.96, *p =* 0.339), suggesting that psychological capital did not significantly moderate the association between microaggressions and physical health. Additionally, the change in R^2^ due to the interaction term was ΔR^2^ = 0.0013, F(1, 563) = 0.92, *p =* 0.339, indicating that adding the interaction term did not significantly improve the model fit. These findings suggest that while both microaggressions and psychological capital independently influence physical health, psychological capital does not significantly moderate the association between microaggressions and physical health. This implies that psychological capital, rather than altering the strength of this association, may function as a direct protective factor against poor physical health outcomes.

## Discussion

### Key findings

1. Mediation Model (X → M → Y): Microaggressions are negatively associated with psychological capital, which in turn worsens physical health.
2. Mediation Model (X → M → Y): Microaggressions are negatively linked to psychological capital, which in turn worsens mental health.
3. Moderation Model (X × W → Y): Psychological capital amplified the association between perceived microaggressions and depressive symptoms, such that higher levels of psychological capital strengthened the positive link between microaggressions and poorer mental health.
4. Moderation Model (X × W → Y): Psychological capital, as a moderator, did not buffer the impact of microaggressions on physical health. However, it remained a significant direct protective factor, independently predicting better physical health outcomes.

The findings of this study demonstrate that perceived microaggressions significantly influence both mental and physical health among migrants in Germany, with psychological capital playing a mediating and, to a lesser extent, a moderating role. Specifically, psychological capital significantly mediated the association between perceived microaggressions and health outcomes. Higher levels of microaggressions were associated with reduced psychological capital, which in turn was linked to poorer mental and physical health outcomes. The mediation findings align with the Conservation of Resources (COR) theory [18], which posits that chronic stressors such as microaggressions are linked to the erosion of psychological resources over time.

### Perceived microaggressions and health outcomes

The findings suggest that perceived microaggressions are significantly associated with both mental and physical health. Consistent with prior research, higher levels of perceived microaggressions are associated with poorer health outcomes [3, 5, 38, 39]. For instance, Sanchez et al. [39] conducted a study involving 308 undergraduate students (164 Asian and 152 Latinx) in the United States, revealing that those reporting higher frequencies of racial microaggressions experienced increased psychological distress. Similarly, Choi et al. [3] examined 353 Asian American college students, identifying a strong positive correlation between racial microaggressions and depressive symptoms. This association was further supported by a meta-analysis of 110 studies, which reinforced the link between perceived discrimination and adverse mental health outcomes [5]. Likewise, findings from a study involving African migrants in Germany indicated that perceived microaggressions were strongly associated with reduced quality of life [38].

### The mediating role of psychological capital

This study extends previous findings by illustrating how psychological capital, a composite construct comprising hope, resilience, optimism, and self-efficacy, possibly mediates the association between microaggressions and health outcomes. The significant mediating role of psychological capital in this study suggests that it may serve as a critical resource for migrants navigating microaggressions. The negative association between microaggressions and psychological capital reflects that experiences of microaggression may undermine an individual's ability to maintain resilience, optimism, hope, and self-efficacy. This aligns with COR theory, which posits that limited resources are related to greater vulnerability to stressors and their consequences. Furthermore, the finding that psychological capital partially mediates the association implies that other factors, such as social support and acculturation strategies, may also contribute to the observed health disparities.

This mediation pathway suggests that efforts to bolster psychological capital could be a valuable intervention strategy to mitigate the adverse effects of microaggressions. Such interventions may include resilience training, optimism enhancement, and programs designed to improve hope and self-efficacy among migrants. Furthermore, acknowledging and validating experiences of microaggressions within supportive environments may help preserve and even strengthen psychological resources.

### The moderating role of psychological capital

Unlike the mediation findings, the moderation analysis produced a more complex pattern. Psychological capital significantly moderated the association between microaggressions and mental health (depressive symptoms), but not physical health (somatic complaints). Interestingly, the interaction indicated an amplifying effect rather than a buffering one: among individuals with higher levels of psychological capital, perceived microaggressions were more strongly associated with depressive symptoms. At the same time, psychological capital exerted a robust main effect, with higher levels consistently linked to fewer depressive symptoms overall. This paradox suggests that while psychological resources generally protect against depression, they may also intensify the psychological toll of microaggressions under certain conditions. One possible explanation is that individuals with higher psychological capital may hold stronger expectations of fairness, inclusion, and social reciprocity; when these expectations are violated through discriminatory encounters, the psychological impact may be amplified [40, 41].

Greater resilience, optimism, and self-efficacy may also encourage migrants to engage more actively in social and professional environments, which may inadvertently increase exposure to subtle forms of discrimination [20]. In addition, recent work on “resilience fatigue” suggests that constant reliance on personal resources to cope with ongoing adversity can itself become taxing over time, leading to heightened vulnerability to stressors [42, 43]. Although psychological capital is typically conceptualised as protective, one plausible explanation for the observed pattern is that higher psychological capital (e.g., greater self-efficacy and hope) may increase attunement to unfair treatment and elevate expectations of being treated justly, such that microaggressions are appraised as more salient or more violating. Empirical work shows that discrimination is linked to depressive symptoms partly through maladaptive cognitive and anticipatory processes, including brooding rumination and vigilance: for example, brooding has been shown to mediate the association between perceived discrimination and depressive symptoms among racial and ethnic minority emerging adults [44], and race-related vigilance has been associated with higher depressive symptoms alongside discrimination exposure [45]. Relatedly, discrimination is robustly associated with poorer mental health across studies, with evidence that stress-related psychological processes are central pathways [5]. From this perspective, individuals with higher psychological capital may be more likely to interpret microaggressions as injustice-based expectancy violations, which could intensify self-focused cognitive processing (e.g., rumination) or sustained vigilance, thereby amplifying depressive symptoms. This interpretation is also consistent with evidence linking discrimination-related appraisals of injustice to depressive symptomatology [46] and with findings that discrimination prospectively predicts increases in depressive symptoms over time [47].

Together, these frameworks highlight that psychological capital may not uniformly buffer stress but can, under specific contexts, exacerbate the negative consequences of microaggressions.

The absence of a moderation effect for physical health suggests that psychological resources do not function as protective factors in mitigating physiological stress responses, such as somatic complaints or chronic pain. This aligns with prior research indicating that psychological capital and related resources (e.g., optimism and resilience) are more strongly associated with emotional regulation and cognitive coping than with physical health outcomes [14].

An additional interpretive consideration concerns the cross-cultural validity of psychological capital in diverse migrant populations. Although the psychological capital questionnaire (PCQ) was originally developed within Western organisational settings, the PCQ (and its short form, the CPC-12) has been validated in some non-Western contexts, including South Africa [48]. Psychometric reviews, however, indicate mixed cross-cultural findings, with some subscales, particularly optimism and resilience, showing less stable performance across different cultural or linguistic groups [49]. These inconsistencies suggest that cultural context may shape how psychological capital is expressed and measured among migrant populations, which is an important consideration when interpreting the moderation effect observed in this study.

From a theoretical perspective, these findings highlight the importance of distinguishing between psychological and physiological outcomes when evaluating the role of psychosocial resources. Psychological capital may foster adaptive coping strategies, positive reappraisal, and emotion regulation, which are closely linked to mental health. However, such resources may not sufficiently offset the cumulative physiological impact of chronic stressors, nor do they uniformly protect against the harmful effects of microaggressions. Future research should investigate why, in specific contexts, higher psychological capital may exacerbate vulnerability to microaggressions, for instance, through heightened expectations of fairness or increased social engagement that increases exposure to discriminatory encounters.

### Length of stay and mental health

The negative correlation between length of stay in Germany and poorer mental health suggests that migrants who have resided longer in the host country report slightly better mental health outcomes. This aligns with the idea that, over time, migrants may gradually adapt to their social environment, develop coping strategies, and establish stronger social networks, which can buffer the impact of stressors. Prior studies have shown that longer residence is often associated with improved familiarity with language, culture, and institutional systems, which in turn can facilitate psychological adjustment and reduce vulnerability to depressive symptoms [50, 51]. For the current sample, this finding may reflect processes of adaptation and resilience: while initial years in Germany might be marked by uncertainty, exclusion, or high stress, over time migrants may regain psychological resources such as hope, resilience, and optimism, thereby improving their mental health. Nevertheless, it is essential to acknowledge that these gains may not be uniform across all migrant groups, as factors such as cultural distance, socioeconomic integration, and exposure to discrimination continue to shape long-term outcomes.

### Theoretical and practical implications

The findings of this study highlight that perceived microaggressions co-occur with reduced psychological capital, a pattern that is also associated with poorer well-being among migrants. In line with the Conservation of Resources (COR) theory [18], chronic exposure to microaggressions is associated with lower levels of key psychological resources—hope, resilience, optimism, and self-efficacy, which in turn are linked to poorer mental and physical health. While psychological capital buffers mental health outcomes, it is less effective for physical health, suggesting a need for targeted interventions that address these distinctions. According to social capital theory [10] and resilience theory [9], psychological capital plays a crucial role in mitigating the psychological impacts of microaggressions. Programs that enhance coping skills, resilience, optimism, and hope may help reduce adverse effects on mental health. Moreover, acknowledging and validating microaggression experiences is essential to avoid harm from gaslighting or victim-blaming [52]. Supportive environments that validate these experiences can empower individuals and enhance their coping capacity.

#### Policy implications

The findings of this study underscore the urgent need for comprehensive anti-discrimination policies that address not only overt acts of discrimination but also subtle and recurring microaggressions. Institutional guidelines in healthcare, education, and employment should explicitly recognise microaggressions as harmful and include transparent reporting and accountability mechanisms. Importantly, *promoting inclusivity* should not be equated with assimilation into majority cultural norms. Rather, inclusivity must be understood as a dual strategy: (a) fostering openness and acceptance within the majority culture toward migrants, and (b) ensuring that migrants have the right and the institutional support to maintain their cultural identities. Policies that explicitly value cultural diversity as a resource, while reducing barriers to participation and access to care, can both reduce exposure to microaggressions and preserve psychosocial capital. In addition, expanding culturally sensitive and accessible mental health services and embedding resilience- and resource-building programs into public health initiatives can further mitigate the adverse health effects of microaggressions and reduce disparities among migrant populations.

#### Practical implications

At the practice level, professionals across healthcare, education, and social work should receive systematic training in cultural competence, anti-racism, and recognising microaggressions. Such training should equip practitioners not only to identify and address subtle discrimination but also to validate the cultural identities of the people they serve. Promoting inclusivity in practice, therefore, requires both encouraging acceptance within the majority culture and providing safe and affirming spaces where migrants can sustain their cultural belonging without pressure to assimilate. Interventions aimed at enhancing psychosocial capital, such as resilience training, optimism and self-efficacy workshops, cognitive-behavioural strategies, and peer support groups, are more effective when they are delivered in environments that respect and affirm cultural identities. Together, these measures reduce the psychosocial burden of microaggressions, strengthen psychological resources, and contribute to improved health outcomes and stronger social cohesion within Germany’s diverse population.

### Limitations

Several limitations of this study should be acknowledged. First, its cross-sectional design prevents causal inferences about the associations between perceived microaggressions, psychosocial capital, and health outcomes. Although we tested mediation and moderation models, these findings should be interpreted as possible explanatory mechanisms rather than causal pathways. Longitudinal studies are needed to confirm directionality and assess changes over time.

Second, the study relied on self-report measures, which may be subject to recall and social desirability biases. While validated instruments were employed, participants’ perceptions may not fully capture their actual health status or the frequency of microaggressions.

Third, although the sample was diverse, it may not be fully representative of all migrant groups in Germany. Factors such as legal status, language proficiency, and socioeconomic background could influence both the experience of microaggressions and access to psychosocial resources, but were not fully accounted for in this study. Moreover, a substantial proportion of our sample consisted of asylum applicants, refugees, and individuals originating from conflict-affected regions (e.g., the Middle East, Ukraine/Eastern Europe). In this study, we did not control for pre-migration trauma or post-traumatic stress, which are strong predictors of somatic complaints and depressive symptoms. Thus, it is difficult to disentangle the unique contribution of current microaggressions from the potential influence of prior traumatic experiences.

Fourth, we did not conduct detailed subgroup analyses by gender or cultural background. Prior research indicates that migrant women may experience unique stressors and health risks, and cultural distance is closely linked to experiences of discrimination and health disparities. These aspects were beyond the scope of the current analysis but represent important directions for future research.

Physical health was assessed using a reduced subset of six items from the PHQ-15 rather than the full validated instrument. Although this approach allowed for a parsimonious assessment of somatic symptom burden and demonstrated acceptable internal consistency, the subset has not been independently validated as a standalone measure. As a result, construct validity may be reduced compared to the full PHQ-15, and findings related to physical health should be interpreted with caution. Future research should replicate these findings using the complete PHQ-15 or other fully validated measures of physical health.

Finally, while psychosocial capital was examined as both a mediator and a moderator, other potentially relevant mechanisms—such as social support, acculturation strategies, or cultural identity- were omitted. Future research should integrate these additional factors and consider the interplay between individual-level and systemic influences to provide a more comprehensive understanding of how microaggressions affect the health and well-being of migrants.

## Conclusion

This study demonstrates that perceived microaggressions negatively impact the mental and physical health of migrants in Germany, primarily through the erosion of psychological capital. Notably, psychological capital amplified rather than buffered the association between microaggressions and depressive symptoms, indicating that higher psychological capital strengthened the link between microaggressions and poorer mental health. Psychological capital did not moderate physical health outcomes, although it remained a significant direct predictor of better physical well-being. This disparity underscores the need for interventions that target psychological capital specifically while also addressing structural barriers to healthcare access and inclusion. The findings suggest that promoting psychological capital through resilience training, culturally sensitive counselling, and validation of lived experiences could mitigate mental health disparities among migrants. However, the lack of protective effects for physical health indicates that psychological resources alone are insufficient; comprehensive strategies addressing healthcare access, institutional bias, and broader social inequalities are essential.

Effective anti-discrimination policies in Germany must explicitly recognise microaggressions as harmful rather than minimising or overlooking their impact. Institutions should implement practical mechanisms for addressing microaggressions alongside efforts to create inclusive and supportive environments that empower migrants to maintain psychological well-being despite ongoing challenges. Future research should employ longitudinal designs to investigate how microaggressions and psychological capital interact over time, particularly in relation to physical health outcomes. Examining the interplay between personal resources and structural barriers will be crucial for developing more comprehensive and impactful interventions that improve the health and integration of migrants in Germany.

## Supplementary Information

Supplementary Material 1.

## Data Availability

The data included in this report are available upon request to the corresponding author,** AA.

## Appendix

**Table 6:** Differences in poor mental health, perceived microaggressions, poor physical health, and psychological outcomes by migrant origin

Migrational Background	Mental Health			Perceived Microaggression			Physical Health			Hope			Self Efficacy			Resilience			Optimism			Psychological Capital		
	Mean	SD	*p*	Mean	SD	*p*	Mean	SD	*p*	Mean	SD	*p*	Mean	SD	*p*	Mean	SD	*p*	Mean	SD	*p*	Mean	SD	*p*
Eastern Europe	9.58	6.43	.35	**8.85**	**7.10**	**.00**	3.84	2.62	.36	**8.92**	**1.91**	**.04**	8.65	1.87	.39	8.61	1.86	.15	9.64	1.95	.27	35.75	5.98	.42
Northern Europe	9.57	7.79	.46	9.64	8.09	.09	3.25	3.41	.25	8.21	2.01	.20	8.50	2.18	.37	8.79	2.26	.48	9.54	2.54	.50	35.08	7.47	.39
Central Europe	**6.89**	**5.03**	**.00**	**9.27**	**9.71**	**.00**	3.27	3.19	.15	8.80	1.78	.33	8.71	1.67	.46	8.83	1.74	.40	9.89	1.89	.14	36.23	5.72	.28
Southern Europe	10.73	7.05	.14	13.56	7.43	.22	4.44	3.21	.14	8.79	1.87	.32	8.71	1.57	.47	8.48	1.65	.15	9.50	1.89	.44	35.48	5.86	.42
Western Europe	10.76	7.74	.23	13.15	7.49	.41	5.14	4.45	.16	8.20	2.31	.18	8.10	2.34	.13	8.48	1.72	.24	9.05	2.42	.12	33.80	7.89	.08
North America	11.50	6.32	.15	13.29	9.58	.40	3.58	3.09	.35	9.00	1.96	.25	8.36	2.82	.33	**7.79**	**1.81**	**.03**	9.43	2.31	.42	34.57	7.50	.25
South/Central America	9.53	6.48	.41	10.94	7.41	.08	4.41	3.46	.18	8.89	1.67	.23	8.78	1.76	.38	9.03	1.54	.19	**10.14**	**1.97**	**.03**	36.83	5.57	.11
Middle East/North Africa	**11.10**	**7.00**	**.02**	**15.62**	**7.68**	**.00**	3.76	3.34	.30	8.59	1.84	.33	8.66	1.60	.44	8.85	2.05	.30	9.59	1.83	.38	35.70	5.88	.46
Sub-Saharan Africa	9.37	6.41	.17	**15.13**	**6.32**	**.00**	3.51	2.91	.21	8.78	1.67	.17	8.81	1.73	.16	8.94	1.92	.07	**9.28**	**1.87**	**.02**	35.82	5.89	.34
Russia/Central Asia	9.60	5.57	.42	11.35	7.63	.06	4.43	2.92	.10	8.79	1.92	.28	8.51	1.77	.21	8.71	2.00	.42	9.84	2.06	.09	35.85	6.45	.38
East Asia	9.11	4.36	.19	12.89	7.91	.45	4.27	3.13	.27	8.49	1.87	.27	9.08	2.10	.09	8.97	2.06	.24	**10.08**	**1.92**	**.04**	36.62	6.71	.16
South Asia	9.83	6.16	.45	13.10	7,53	.31	3.85	3.21	.41	**8.13**	**1.97**	**.00**	8.71	2.04	.45	8.85	1.88	.29	9.24	2.14	.06	34.93	6.35	.11
Southeast Asia/Australia	9.81	5.43	.48	12.72	7.31	.50	3.48	2.58	.17	8.51	1.52	.27	8.53	1.60	.28	**8.32**	**1.83**	**.05**	9.45	1.74	.37	34.81	5.33	.17
Missing	10.33	6.08	.38	14.27	5.62	.25	4.20	4.32	.42	8.92	2.47	.32	9.00	1.65	.28	9.25	1.86	.18	9.67	2.15	.41	36.83	6.51	.25

## Abbreviations

AMOS: Analysis of Moment Structures
BMS-9: BeLiv Microaggression Scale – Short Version (9 items)
BMS-27: BeLiv Microaggression Scale – Long Version (27 items)
CFI: Comparative Fit Index
COR: Conservation of Resources (Theory)
DSM-IV: Diagnostic and Statistical Manual of Mental Disorders, 4th Edition
FIML: Full Information Maximum Likelihood
HC3: Heteroskedasticity-Consistent Standard Errors, Type 3
IFI: Incremental Fit Index
ISCED: International Standard Classification of Education
JN: Johnson-Neyman (Technique)
PCQ-12: Psychological Capital Questionnaire – 12 Items
PHQ-9: Patient Health Questionnaire – Depression Module
PHQ-15: Patient Health Questionnaire – Somatic Symptoms Module
RMSEA: Root Mean Square Error of Approximation
SEM: Structural Equation Modelling
SD: Standard Deviation
SRMR: Standardised Root Mean Square Residual
TLI: Tucker-Lewis Index
VIF: Variance Inflation Factor
